# The Prevalence of Dyslipidemia among Jordanians

**DOI:** 10.1155/2018/6298739

**Published:** 2018-10-28

**Authors:** Mousa Abujbara, Anwar Batieha, Yousef Khader, Hashem Jaddou, Mohammed El-Khateeb, Kamel Ajlouni

**Affiliations:** ^1^The National Center (Institute) for Diabetes, Endocrinology and Genetics (NCDEG)/The University of Jordan, Jordan; ^2^Department of Community Medicine, Jordan University of Science and Technology, Irbid, Jordan; ^3^Jordan University of Science and Technology (JUST), Irbid, Jordan; ^4^The Jordan University of Science and Technology (JUST), Irbid, Jordan

## Abstract

**Background:**

Dyslipidemia is one of the major modifiable risk factors for the development of cardiovascular disease and type two diabetes mellitus. Knowing the current prevalence of dyslipidemia is an important step for increasing awareness of the problem and for proper planning of health programs for prevention of its negative clinical effects.

**Methods:**

A national population based household sample was selected from north, middle, and south regions of Jordan in 2017. A total sample of 4,056 aged between 18 and 90 years were included. Selected individuals were interviewed using a structured questionnaire and fasting blood samples were collected for biochemical measurements.

**Results:**

The prevalence rates of hypercholesterolemia, hypertriglyceridemia, high LDL, and low HDL were 44.3%, 41.9% 75.9%, and 59.5%, respectively. Hypercholesterolemia in Jordan almost doubled from 23.0% in 1994 to 44.3% in 2017, and hypertriglyceridemia increased from 23.8% in 1994 to 41.9% in 2017. All lipid abnormalities decreased after the age of 60 years. Hypertension, diabetes, and obesity were all independently associated with hypercholesterolemia and hypertriglyceridemia.

**Conclusions:**

Results of this study show that dyslipidemia is a widely prevalent health problem among adult Jordanian population and that the problem has substantially increased since 1994. Encouraging healthy lifestyle and healthy diet are the basis for prevention of dyslipidemia.

## 1. Introduction

Noncommunicable diseases (NCDs) are considered the leading cause of death in Jordan, with 38% of deaths attributed to cardiovascular diseases (CVDs) [1]. With the rapid lifestyle changes and socioeconomic development, the prevalence of NCDs has markedly increased over the past years in Jordan [2]. It was estimated that between 1 and 3 million persons in Jordan will have hypertension, high blood cholesterol, or diabetes by 2050 [3]. Dyslipidemia is one of the major modifiable risk factors for the development of cardiovascular diseases, atherosclerosis, and type two diabetes mellitus [4]. Guidelines for screening and treating dyslipidemia indicate that proper management of lipids is both lifesaving and cost-effective. Furthermore, recent research shows that the association between blood cholesterol level and atherosclerotic cardiovascular diseases is independent of other risk factors and causal associations [5]. Knowing the current prevalence of dyslipidemia is an important step for increasing awareness of the problem and for proper planning of health programs for prevention of the problem and its negative clinical effects. In 1994, a national community-based survey was conducted to estimate the prevalence of dyslipidemia and other cardiovascular risk factors in Jordan [6]. The present study is a follow-up survey using generally a similar methodology, to assess the current prevalence of dyslipidemia in Jordan and to identify its distribution among subgroups of the population.

## 2. Materials and Methods

A cross-sectional community-based survey targeting adults, ≥18 years of age in 2017, was conducted by the National Center for Diabetes, Endocrinology and Genetics (NCDEG), Jordan, Ministry of Health (MoH), and Jordan University of Science and Technology (JUST). A multistage sampling technique was used to represent rural and urban population in the three geographic regions of Jordan (north, middle, and south). As the survey procedures have to take place in health facilities (anthropometric measures, drawing blood samples…etc.). General Health Directors of each of the 12 governorates of Jordan were asked to select one to three health centers that could represent the rest of health centers in each governorate. The selected health centers had to be large enough to provide the needed space for the survey team (25 persons) to perform the required procedures. A team of two field researchers (a male and a female) went door to door to invite a systematic sample of households in the catchment area of the selected center.

The team explained the study protocol and responded to all questions. If they agreed to participate, they were asked to report to the health center at 8:00 am, fasting for ten hours, and to bring all of their medications with them to be checked by the survey team. They were also asked not to take their medications on the day of their visit to the health center. All household members ≥ 18 years of age were eligible to participate in the study. However, only subjects ≥ 25 years of age were included in this analysis. The reason for including only this age group in the analysis is to allow for comparison with the previous national survey and with results from other countries. To encourage participation in the study, visit dates to health centers were negotiated with eligible participants. The study team worked all weekdays (except Fridays) to obtain the highest possible response rate. If a person refused to participate, basic data were collected when possible such as age, gender, education, whether the person was diagnosed with any NCDs, and the reason for nonparticipation. Total response rate was 40% for males and 94% for females. A total sample of 4,056 subjects completed the survey and were included in the analysis.

The survey protocol was approved by the Ethics Committee of the National Center for Diabetes, Endocrinology, and Genetics, Amman, Jordan. A letter of support was obtained from the Minister of Health (MoH) to ensure the cooperation of the staff of the health centers. An informed consent was obtained from all study participants. Study participants were allowed to decline participation in any part of the study. All study procedures were carried out free of charge for the participants. Data were treated with high confidentiality and used only for scientific purposes.

All field researchers attended a two-day training workshop, prior to data collection. Training included explaining the study purpose and procedures, proper interview skills, and standard methods of anthropometric measurements. Health workers responsible for obtaining blood samples were trained on the standard aseptic technique for obtaining blood samples.

Selected individuals were interviewed using a structured questionnaire that inquired about socio-demographic characteristics, smoking and other variables related to this study. Height was measured without footwear using a portable stadiometer and the reading was recorded to the nearest 1 cm. Weight was measured by a standard weighing scale in light clothes and without footwear with a precision of 100 gm. Body mass index (BMI) was calculated as weight divided by height squared (kg/m^2^). Waist circumference was measured by a nonstretchable tape at the narrowest horizontal plane midway between the iliac crest and lower costal margin with a 1 cm precision. Hip circumference was measured at the widest horizontal plane at the hip level with a precision of 1 cm. Waist to height ratio was calculated as waist circumference divided by height. Three blood samples were drawn from a cannula inserted into the antecubital vein using vacutainer tubes. Collected blood samples were centrifuged within one hour in the field and transported on daily basis in cold boxes from the field to the Central Laboratory of the National Center for Diabetes, Endocrinology, and Genetics in Amman, Jordan, where all laboratory measurements were performed. All biochemical measurements were done by the same team of laboratory technicians using the same method throughout the survey period. Lipids measurements were performed according to the manufacturers' instructions, using COBAS autoanalyzer (Roche Diagnostics, Basel, Switzerland).

## 3. Definitions of Variables

Lipid variables were defined according to the Third Report of the National Cholesterol Education Program (NCEP-Adult Treatment Panel III). Hypercholesterolemia was defined as a serum cholesterol level of ≥ 5.17 mmol/l (200 mg/dl) or on lipid-lowering medication; high low-density lipoprotein (LDL-C) was defined as LDL-C level of ≥ 2.59 mmol/l (100 mg/dl) or on lipid-lowering medication; low high-density lipoprotein (HDL-C) was defined as HDL-C level of ≤ 1.03 mmol/l (40 mg/dl) in men, and ≤ 1.29 mmol/l (50 mg/dl) in women or on lipid-lowering medication. Hypertriglyceridemia was defined as a serum triglyceride (TG) ≥ 1.70 mmoi/l (150 mg/dl) or on lipid-lowering medication. Total cholesterol to HDL-C ratio (TC/HDL ratio) was considered high if the ratio was ≥ 5. Hypertension was defined as systolic blood pressure ≥130 mmHg or diastolic blood pressure >85 mmHg or if the patient was on antihypertensive drugs. Diabetes mellitus was defined as a fasting plasma glucose ≥ 7.0 mmol/l (126 mg/dl) or a HbA1C ≥ 6.5%, or on treatment for diabetes. Smoking was classified into nonsmoker and current smoker (smoked cigarettes daily or occasionally). Participants with BMI between 25 and 29.9 were considered overweight and with BMI of 30 or more were considered obese. Waist circumference was considered abnormal at ≥ 94 cm in men and ≥ 80 in women. Waist-to-height ratio was considered elevated if it was > 0.5.

## 4. Statistical Analysis

Survey data were analyzed using the Statistical Package for Social Sciences (SPSS Version 23). Range and logical checks were used to detect errors in data entry. Detected errors were corrected by returning to the original data forms. A P-value of ≤ 0.05 was considered statistically significant. Comparisons were made between participants with dyslipidemia and the rest of the sample to identify potential associations that were tested for statistical significance using the chi square test for categorical variables. Multivariate logistic regression was used to determine the effect of a given variable while simultaneously controlling for potential confounders. The magnitude of the associations was assessed by the adjusted odds ratio, and the statistical significance was assessed by the Wald chi square.

## 5. Results

This study included 4,056 subjects (70.6% females and 29.4% males) aged between 18 and 90 years with a mean age (SD) of 43.7 (14.2) years. The majority of the subjects were either overweight or obese (74.5%) and 14.5% of them were current smokers. The prevalence rates of hypercholesterolemia, hypertriglyceridemia, high LDL and low HDL were 44.3%, 41.9% 75.9%, and 59.5%, respectively.

Table 1 shows the prevalence of dyslipidemia according to subjects' socio-demographic characteristics. Prevalence of hypercholesterolemia (≥5.17 mmol/l) in females was 44.8% and 43.0% in males. Hypertriglyceridemia (≥ 1.70 mmol/l) was 36.5% in females significantly lower than in males (54.6%). Study participants showed no statistical difference in prevalence of low HDL-C and high LDL-C between male and female participates. On the other hand, the prevalence of TC/HDL ratio >5 in males was double that in females and the difference was statistically significant. The prevalence of hypercholesterolemia and hypertriglyceridemia reached its peak at the age group of 50-59 years then decreased again, while the prevalence of low HDL-C increased until the age of 39 years and it plateaued at 40 to 69 years to decrease at age 70. The prevalence of low LDL-C reached its peak between 40 to 49 years then decreased gradually among the older age groups. Similar significant change in the prevalence of TC/HDL ratio >5 was noticed where the prevalence increased until the age group of 50 to 59 years then decreased in the older age groups. Prevalence of hypercholesterolemia and hypertriglyceridemia was significantly higher among obese and overweight participants than normal BMI participants where the prevalence of hypercholesterolemia and TG was 50.6% and 52.7%, respectively, in obese participants. Also, the highest prevalence of low HDL-C and high LDL-C and TC/HDL ratio >5 was noticed among obese participants. Likewise, participants with waist-to-height ratio >0.5 had hypercholesterolemia, hypertriglyceridemia, low HDL-C, high LDL-C, and TC/HDL ratio >5 prevalence significantly higher than participants with waist-to-height ratio ≤ 0.5. The difference in prevalence of dyslipidemias was not statistically significant in different regions in Jordan except for hypertriglyceridemia which was lower in participants living in the middle region in comparison with participants living in other regions. Study results showed no significant difference in hypercholesterolemia between smokers and nonsmokers, while the prevalence of hypertriglyceridemia in smokers (53.9%) was significantly higher than in nonsmokers (39.7%). Low HDL-C was significantly higher in smokers than nonsmokers, while high LDL-C was not significantly different between smokers and nonsmokers. TC/HDL ratio >5 was significantly more prevalent in smokers than in nonsmokers. On bivariate analysis, the prevalence of hypercholesterolemia and high LDL-C was almost the same in diabetic and nondiabetic subjects and in hypertensive and normotensive subjects. While, hypertriglyceridemia, low HDL-C, and TC/HDL ratio >5 prevalence were all significantly higher in diabetic subjects when compared with nondiabetics and in hypertensive subjects when compared with normotensive subjects.

Tables 2–5 shows multivariate regression findings for hypercholesterolemia, hypertriglyceridemia, low HDL-C, and high LDL-C, respectively.  Table 2 shows that hypercholesterolemia was significantly associated with hypertension (Odds Ratio (OR)= 1.37, 95% Confidence Interval (CI)= 1.15-1.63) and diabetes (OR=1.25, 95% CI=1.04-1.50). Age, overweight, and obesity were all significantly associated with increased risk of hypercholesterolemia.  Table 3 shows that hypertriglyceridemia was significantly associated with hypertension (OR=1.36, 95% CI=1.13-1.62), diabetes (OR=1.68, 95% CI=1.39-2.04), and cigarette smoking (OR=1.53, 95% CI=1.24-1.89). Similarly, overweight subjects were more likely to have hypertriglyceridemia (OR=2.01, 95% CI=1.61-2.50) and obese subjects were also more likely to have hypertriglyceridemia (OR=2.89, 95% CI=2.33-3.58) compared with subjects with normal BMI. Study subjects in 40-49, 50-59, and 60-69 age groups were more likely to have hypertriglyceridemia compared to subjects less than 40 years old, whereas female gender (OR=0.49, 95% CI=0.42 - 0.57), being single and living in the middle or south region were all independent protective factors from hypertriglyceridemia.  Table 4 shows that low HDL-C was significantly associated with hypertension (OR=1.31, 95% CI=1.10 – 1.55), diabetes (OR=1.47, 95% CI=1.21 – 1.77), waist circumference >94 cm in men or >80 cm in women (OR=1.48, 95% CI=1.21-1.81), and obesity (OR=1.75, 95% CI=1.41-2.22), while female gender was an independent protective factor from low HDL-C (OR=0.83, 95% CI=0.71 – 0.96).  Table 5 shows that high LDL-C was significantly associated with hypertension (OR=1.35, 95% CI=1.09-1.67), diabetes (OR=1.66, 95% CI=1.33-2.07), and abnormal waist circumference (OR=1.63, 95% CI=1.35-1.96). In addition, 40-49-, 50-59-, 60-69-, and a 70-year age groups were significantly more likely to have high LDL-C compared to subjects less than 40 years old. Also, overweight and obese subjects were more likely to have high LDL-C compared to subjects with normal BMI.

## 6. Discussion

Results of this study show that dyslipidemia is a widely prevalent health problem affecting adult Jordanian population and putting them at an enormous risk for CVD [7, 8]. To the best of our knowledge, this study is one of a few community-based studies conducted in Jordan over the past 23 years. The prevalence of hypercholesterolemia in Jordan increased from 23.0% in 1994 to 44.3% in 2017, and the prevalence of hypertriglyceridemia increased from 23.8% in 1994 to 41.9% in 2017 [6]. This marked increase could be explained by lifestyle changes and by changes in food consumption pattern. However, better survival of subjects with dyslipidemia may have contributed to this increase.

A remarkable finding of this study was that all types of lipid abnormalities decrease after the age of 60 years. Streja et al. reported similar findings in a chapter about management of dyslipidemia in the elderly [9]. A possible explanation for this finding is that many people with dyslipidemia might die before the age of 60, leaving only a few surviving to this age. Attrition or survival bias can decrease associations between harmful exposures and illnesses of aging [9].

Previous studies from our region showed variable results. In a study of Kuwaiti adults, 33.7% of men and 30.6% of women were reported to have hypercholesterolemia. Another study in Oman reported hypercholesterolemia prevalence of 33.6% [10, 11]. On the other hand, a higher prevalence of hypercholesterolemia was reported from Saudi Arabia (54%) [12] while an Iranian meta-analysis reported a hypercholesterolemia prevalence of 41.6% which is very close to our figure of 44.3% [13]. Differences in study design, dietary habits, lifestyles, and accessibility to health care may account for much of the variations in the prevalence of hypercholesterolemia.

Hypertriglyceridemia prevalence in the current study population (41.9%) was close to that reported from Saudi Arabia (40.3%) [12], and Iran (46.0%) [13].

The present study showed the independent risk factors of the different types of dyslipidemia by multivariate analysis. Diabetes mellitus and hypertension were independent risk factors for hypercholesterolemia. A study from Saudi Arabia also reported same association between diabetes mellitus, hypertension, and hypercholesterolemia [14]. Similarly, obesity was an independent risk factor for hypercholesterolemia in the current study, in an Ethiopian national survey and in a study from Beijing China [15, 16]. Additionally, hypertriglyceridemia was independently associated with diabetes mellitus. A similar association between hypertriglyceridemia and HbA1c was reported in a Russian study [17].

An interesting finding was that living in northern region of Jordan was an independent risk factor for hypertriglyceridemia. We have no clear explanation for this finding which may need further investigation.

The prevalence of low HDL-C and prevalence of high LDL-C were positively and independently associated with obesity, increased waist circumference, diabetes, and hypertension. This is consistent with a recent study in Turkey [18]. There have been a lot of changes regarding the optimal management of dyslipidemia in the last few years. The American Association of Clinical Endocrinologists and American College of Endocrinology 2017 guidelines (AACE 2017) for management of dyslipidemia and prevention of cardiovascular disease encourage physicians to provide personalized goals of lipid levels to their patients and states that the 10-year risk of a coronary event need to be assessed in order to provide such personalized lipid management [5].

This study had two main limitations: firstly, it was a cross-sectional survey, in which the risk factors and dyslipidemia were assessed simultaneously. Consequently, it is not possible to judge whether dyslipidemia was present before or after the proposed risk factors; i.e., the temporal sequence from cause to effect could not be established. Secondly, our associations are based on prevalence rather incidence. Prevalence is affected by duration of the disease which may in turn be related to health care; thus differences in prevalence may merely reflect differences in survival rather differences in incidence.

In conclusion, it was clear that dyslipidemia is high in Jordan which may put a high burden on the already strained health services and leads to substantial increase in health related costs. Prevention of dyslipidemia is critical to achieve the goal of halting the rise in cardiovascular diseases. At the community level, encouraging healthy lifestyle, healthy dietary habits, and physical exercise are the cornerstone for prevention of dyslipidemia. Screening of high risk groups such as obese people is recommended.

## Figures and Tables

**Table 1:** Prevalence of dyslipidemia by selected variables, Jordan, 2017.

**Variables**	**Hypercholesterolemia** ≥5.17 mmol/l n (%)	**Hypertriglyceridemia ** ≥1.70 mmol/l n (%)	**Low HDL-C** F ≤1.29 mmol, M ≤1.03 mmol/l n (%)	**High LDL-C** ≥2.59 mmol/l n (%)	**TC/HDL ratio** >5 n (%)
**Overall **	1780 (44.3)	1683 (41.9)	2391 (59.5)	3049 (75.9)	1335 (33.2)

**Gender **					
Male	509 (43.0)	647 (54.6)	732 (61.8)	883 (74.6)	619 (52.3)
Female	1271 (44.8)	1036 (36.5)	1659 (58.5)	2166 (76.4)	716 (25.3)

P-value	0.274	0.000	0.052	0.218	0.000

**Age (years)**					
25-29	83 (29.5)	58 (20.6)	120 (42.7)	179 (63.7)	44 (15.7)
30-39	324 (40.5)	281 (35.1)	449 (56.1)	611 (76.4)	219 (27.4)
40-49	562 (52.7)	516 (48.4)	665 (62.4)	907 (85.2)	419 (39.3)
50-59	467 (55.7)	457 (54.5)	550 (65.6)	696 (83.1)	377 (45.0)
60-69	208 (51.9)	245 (61.1)	269 (67.1)	314 (78.3)	178 (44.4)
≥ 70	75 (41.9)	78 (43.6)	104 (58.1)	132 (73.7)	62 (34.6)

P-value	0.000	0.000	0.000	0.000	0.000

**BMI (kg/m** ^**2**^ **)**					
Normal	249 (31.0)	166 (20.7)	364 (45.3)	499 (62.1)	142 (17.7)
Overweight	602 (46.4)	548 (42.3)	735 (56.7)	1030 (79.4)	446 (34.4)
Obese	916 (50.6)	954 (52.7)	1243 (68.7)	1467 (81.1)	737 (40.7)

P-value	0.000	0.000	0.000	0.000	0.000

**Waist to height ratio**					
Normal	954 (40.2)	787 (33.1)	1246 (52.5)	1714 (72.2)	620 (26.1)
Abnormal	821 (50.4)	890 (54.6)	1135 (69.7)	1323 (81.2)	711 (43.6)

P-value	0.000	0.000	0.000	0.000	0.000

**Geographical region **					
North	579 (44.7)	602 (46.5)	785 (60.6)	991 (76.5)	441 (34.1)
Middle	784 (44.4)	648 (36.7)	1030 (58.4)	1316 (74.6)	559 (31.7)
South	417 (43.4)	433 (45.1)	576 (60.0)	742 (77.3)	335 (34.9)

P-value	0.809	0.000	0.433	0.251	0.175

**Marital status **					
Married	1452 (47.2)	1422 (46.2)	187 (61.0)	2438 (79.2)	1131 (36.8)
Single	141 (23.7)	88 (14.8)	298 (50.0)	331 (55.5)	74 (12.4)
Divorced or widow	180 (53.9)	164 (49.1)	206 (61.7)	271 (81.1)	122 (36.5)

P-value	0.000	0.000	0.000	0.000	0.000

**Smoking **					
Smoker	258 (44.4)	313 (53.9)	420 (72.4)	444 (76.6)	316 (54.5)
Non-smoker	1513 (44.3)	1356 (39.7)	1957 (57.2)	2590 (75.8)	1009 (29.5)

P-value	0.945	0.000	0.000	0.678	0.000

**Hypertension **					
Yes	438 (46.3)	563 (59.5)	664 (70.2)	729 (77.1)	414 (43.8)
No	1329 (43.7)	1103 (36.3)	1704 (56.0)	2296 (75.5)	908 (29.9)

**P-value **	0.160	0.000	0.000	0.000	0.000

**Diabetes mellitus **					
Yes	353 (46.3)	485 (63.6)	547 (71.9)	564 (74.1)	341 (44.8)
No	1420 (43.8)	1186 (36.6)	1832 (56.5)	2471 (76.3)	985 (30.4)

**P-value **	0.212	0.000	0.000	0.212	0.000

**Table 2:** Adjusted odds ratios and their 95% Cl for Hypercholesterolemia using multivariate logistic regression, Jordan 2017.

**Variable**	**Adjusted OR**	**95% CI**
**Gender **		
Male	1	
Female	1.17	1.00-1.35

**Age (years)**		
< 40	1	
40-49	2.37	1.99-2.82
50-59	2.87	2.36-3.49
60-69	2.73	2.11-3.53
≥ 70	1.92	3.62-6.67

**BMI **		
Normal	1	
Overweight	1.63	1.34-1.97
Obese	1.77	1.46-2.14

**Hypertension **		
No	1	
Yes	1.37	1.15-1.63

**Diabetes **		
No	1	
Yes	1.25	1.04-1.50

**Table 3:** Adjusted odds ratios and their 95% Cl for Hypertriglyceridemiausing multivariate logistic regression, Jordan, 2017.

**Variable**	**Adjusted OR**	**95% CI**
**Gender **		
Male	1	
Female	0.49	0.42 - 0.57

**Age (years)**		
< 40	1	
40-49	1.65	1.37-1.98
50-59	1.64	1.33-2.02
60-69	1.84	1.40-2.42
≥ 70	0.44	0.60-1.24

**BMI **		
Normal	1	
Overweight	2.01	1.61-2.50
Obese	2.89	2.33-3.58

**Hypertension **		
No	1	
Yes	1.36	1.13-1.62

**Diabetes **		
No	1	
Yes	1.68	1.39-2.04

**Marital status **		
Married	1	
Single	0.41	0.31-0.53
Divorced or widow	1.06	0.82-1.37

**Geographical region **		
North	1	
Middle	0.68	0.58-0.80
South	0.77	0.64-0.93

**Smoking **		
Non-smoker	1	
Smoker	1.53	1.24-1.89

**Table 4:** Adjusted odds ratios and their 95% Cl for low HDL-C using multivariate logistic regression, Jordan 2017.

**Variable**	**Adjusted OR**	**95% CI**
**Gender **		
Male	1	
Female	0.83	0.71 – 0.96

**BMI **		
Normal	1	
Overweight	1.18	0.96- 1.46
Obese	1.75	1.41-2.22

**Waist circumference**		
Normal	1	
Increased	1.48	1.21-1.81

**Hypertension **		
No	1	
Yes	1.31	1.10 – 1.55

**Diabetes **		
No	1	
Yes	1.47	1.21 – 1.77

**Table 5:** Adjusted odds ratios and their 95% Cl for high LDL-Cusing multivariate logistic regression, Jordan 2017.

**Variable**	**Adjusted OR**	**95% CI**
**Gender **		
Male	1	
Female	1.19	1.00 – 1.43

**Age (years)**		
< 40	1	
40-49	2.90	2.33-3.60
50-59	2.76	2.16- 3.52
60-69	2.29	1.68-3.12
≥ 70	1.95	1.32 – 2.87

**BMI **		
Normal	1	
Overweight	2.07	1.69-2.54
Obese	2.11	1.73-2.58

**Waist circumference**		
Normal	1	
Increased	1.63	1.35-1.96

**Hypertension **		
No	1	
Yes	1.35	1.09-1.67

**Diabetes **		
No	1	
Yes	1.66	1.33-2.07

## Data Availability

The SPSS data used to support the findings of this study are available from the corresponding author upon request.
